# Rice Yellow Mottle Virus (RYMV): A Review

**DOI:** 10.3390/v16111707

**Published:** 2024-10-31

**Authors:** Linda Appianimaa Abrokwah, Stephen Kwame Torkpo, Guilherme da Silva Pereira, Allen Oppong, John Eleblu, Justin Pita, Samuel Kwame Offei

**Affiliations:** 1 Department of Crop Science, School of Agriculture, University of Ghana, Legon P.O. Box LG 68, Ghana; l.abrokwah@cropsresearch.org (L.A.A.); soffei@ug.edu.gh (S.K.O.); 2 CSIR-Crops Research Institute, Kumasi-Ghana P.O. Box 3785, Ghana; a.oppong@cropsresearch.org; 3 Forest and Horticultural Crops Research Centre-Kade, School of Agriculture, College of Basic and Applied Sciences, University of Ghana, Legon P.O. Box LG 25, Ghana; 4 Department of Agronomy, Universidade Federal de Viçosa (UFV), Viçosa 36570-900, Brazil; g.pereira@ufv.br; 5 West Africa Centre for Crop Improvement, College of Basic and Applied Sciences, University of Ghana, Legon P.O. Box LG 25, Ghana; jeleblu@ug.edu.gh; 6 Universite Felix Houphouet Boigny, Abidjan 00225, Côte d’Ivoire; justin.pita@wave-center.org

**Keywords:** rice yellow mottle virus, sub-Saharan Africa, constraint, management

## Abstract

Rice (*Oryza* spp.) is mostly grown directly from seed and sown on wet or dry seed beds or usually used as transplants on nursery beds. Among all the economically important viral diseases in the world, rice yellow mottle virus (RYMV) is only prevalent in rice-growing countries in Africa. RYMV has become the main rice production constraint in Africa over the last 20–25 years, causing yield losses of 10 to 100% depending on the age of the plant at the time of infection, degree of varietal susceptibility and the existing climatic conditions. Good agricultural practices and biotechnological tools in the development of improved resistant cultivars have been extensively utilized in controlling the disease. This review focuses on RYMV, its epidemiology, serological and molecular typing, disease management and the way forward for sustainable rice production.

## 1. Introduction

Rice (*Oryza* spp.) is very crucial in tackling food security challenges across the globe [1]. It is currently one of the main staple foods in Africa, with an annual production of 38 million tons; it serves as a basic dietary source for people across the continent [2]. Even though rice is traditionally linked with Asian countries, its consumption is becoming very popular in Africa because of the increase in the human population and urbanization coupled with changes in lifestyle, which demand an increase in rice production [3,4]. In West Africa, rice is the solitary dominant supply of nutrients and is often associated with hospitality and community gatherings. It is the third most important cereal in all of Africa after maize and sorghum. Usually, it forms the basis for lots of traditional dishes and meals; however, it is also used industrially and processed into wine and rice cakes, and the rice straw is used as livestock feed [5].

Rice farming offers substantial employment opportunities across various areas such as irrigation systems, drainage management, and its entire value chain. Moreover, the economic significance of the crop’s production for African nations’ revenues cannot be overstated. Given its increasing role in consumers’ diets, this crop has become a politically influential commodity, and fluctuations in its price as well as availability significantly impact social stability in many African countries [6]. Since the 2007–2008 food crisis, global rice prices have tripled [7], and they have not fully returned to pre-crisis levels. In sub-Saharan Africa (SSA), its consumption far surpasses the local production capacity. However, with improved rice cultivation systems, this region could reduce its dependency on imports and substantially increase exports.

In sub-Saharan Africa (SSA), rice consumption excessively exceeds production; however, with an efficient system of rice cultivation, the importation of rice would be reduced, and exports would increase considerably. In 2018, it was anticipated that rice consumption in SSA would reach about 33.2 metric tons, out of which 15.5 metric tons was imported, corresponding to 33% of that traded in the world market [8]. According to [9], the import bill was estimated at 6.4 billion USD in 2018, and about 16.6 metric tons of rice was imported into Africa in the 2020/2021 trade year.

Regarding Sustainable Development Goal (SDG) 2, food security is a very important component. Rice is a basic staple for food security and social stability in SSA [10]. Over the past decade, the demand for rice has risen at a rate of 6% per annum [8], making it the commodity with the fastest growth rate in the world and has contributed significantly to global food security [11] during the past half century.

Increased rice production is hampered by unfavorable abiotic and biotic factors [12]. These include low soil fertility (mainly with sub-optimal nitrogen), a high incidence of pests and pathogens causing diseases such as Rice yellow mottle disease (a viral disease leading to the yellowing of leaves, stunted growth, and reduced yield) [13], Rice blast (*Magnaporthe oryzae*, a fungal disease that causes lesions on panicles, leaves and stems, which severely affects yield), and Bacterial leaf blight (*Xanthomonas oryzae* pv. *oryzae*, a bacterial disease causing wilting and drying of leaves, reducing grain quality and yield) [14]. Some diseases caused by pests on the crop include African Rice Gall Midge (*Orseolia oryzivora*, an insect that forms galls on rice stems, stunting plant growth and reducing yield), Rice Stem Borers (e.g., *Sesamia calamistis*, *Chilo* spp., an insect that bores into rice stems, leading to dead hearts and whiteheads, which significantly impact yield), Rice Leaf Rollers (*Cnaphalocrocis medinalis*, an insect that rolls and feeds on rice leaves, reducing the photosynthetic area and plant vigor) [14]. 

Rice yellow mottle disease, caused by rice yellow mottle virus (RYMV), is a major challenge to rice production in Africa. The disease affects rice under all types of cultivation systems including lowland, upland, rainfed and floating mangrove rice [15]. The disease was initially described and named by Bakker [16]. Rice yellow mottle virus primarily infects rice (*Oryza sativa*), but it can also infect several other grass species, including wild rice (*Oryza longistaminata* and *Oryza barthii*). These wild rice species can serve as reservoirs for the virus [17]. Barnyard Grass (*Echinochloa* spp.) is very common in rice fields and can harbor the virus. Cutgrass (*Leersia hexandra*), which is often found in rice-growing regions, and Bermuda Grass (*Cynodon dactylon*) are both known to be potential hosts [18]. Initial variations like Sindano (IR22) and Basmati 217 among others that were introduced into the continent mostly proved to be highly susceptible to the virus [19]. RYMV is widespread across sub-Saharan Africa and affects many rice-producing regions, including West Africa, where countries such as Nigeria, Ghana, Mali, Senegal and Côte d’Ivoire have reported significant outbreaks. Here, the virus is particularly severe in lowland and irrigated rice systems [20]. In Central Africa, RYMV is present in countries like Cameroon, Chad and the Central African Republic, affecting both upland and lowland rice [13]. RYMV infections have been reported in major rice-producing East African countries such as Tanzania, Uganda, Kenya and Madagascar, where it is especially prevalent in irrigated rice fields [16]. Within Southern Africa, Mozambique, Zambia and Zimbabwe have reported occurrences of RYMV, affecting both small-scale and large-scale rice farming [14]. Increased rice cultivation to meet the high demand for consumption across the continent due to the availability of water for sequential plantings throughout the year heightens the spread of RYMV [21]. The widespread distribution and ability to infect multiple hosts make RYMV a significant threat to rice production across SSA.

The infection of the plant can occur at all stages from transplanting to booting. Since booting is when meiosis happens, stresses at this stage may reduce the rice grain yield [22]. Also, depending on the type of rice genotype grown, the RYMV strain type and the time of infection, the heads produce grains that are unfilled, resulting in yield loss, which may range between 10 and 100% [13,23], whereas plants under severe attack may die [5]. 

Although there is a significant relationship between the intensity of symptoms and yield loss, yield loss provides a better assessment of isolates and varietal response to RYMV infection than symptom expression or plant height [17]. RYMV has been consistently very severe in some regions, causing farmers to abandon their fields and plant new ones. Susceptible cultivars have been eliminated by the disease due to its potential to cause unexpected epidemic outbreaks [15,24].

Plants infected by RYMV are mottled and show varying intensities of yellow to orange coloration of the leaves, which could be mistaken for iron toxicity or nitrogen deficiency [25]. Infection is also characterized by stunted growth, sterile flowers and reduced tillering leading to poor panicle exertion, grain discoloration and grain or spikelet sterility [5,18]. 

The threats RYMV poses to food security have garnered significant attention. This review aims to explore the dual nature of RYMV and its agronomic importance. It will also highlight the strategies implemented for sustainable management and the successes achieved. The insights from this review are intended to guide breeding programs, inform the scientific community, and shape national policies for managing RYMV in Africa.

## 2. Epidemiology of Rice Yellow Mottle Virus

RYMV is transmitted by insect vectors through the tripartite connection between plants, insects and viruses and or mechanical movement [26]. The virus is transmitted by several species of beetles, most of which belong to the order Coleoptera and family *Chrysomelidae* [27]. About fifteen species of beetles have been observed as insect vectors for RYMV [28]. This group of insects was the first to be recognized as vectors of RYMV after Bakker discovered the disease. Later, it was observed that their population was on the low compared to the high incidence of the disease. This gave the intuition that other insect vectors could be involved in the transmission of the disease, especially at the seedling stage where they distribute the virus at little to near the ground levels but the possibility of these vectors contaminating the plant was not ruled out [16]. Insects from the order Coleoptera, Orthoptera, Hemiptera, and Diptera are well documented to be effective vectors of RYMV [29]. All these insect vectors of RYMV can be grouped into four according to their morphology and chewing or feeding mouthparts as beetles (e.g., *Sesselia pusilla*, *Chaetocnema pulla*), grasshoppers (e.g., *Conocephalus merumontanus*, *Conocephalus longipennis*), leafhoppers (e.g., *Cofana spectra*, *Nephotettix modulates*) and true flies (e.g., *Diopsis thoracica*) [30].

**Figure 1:**
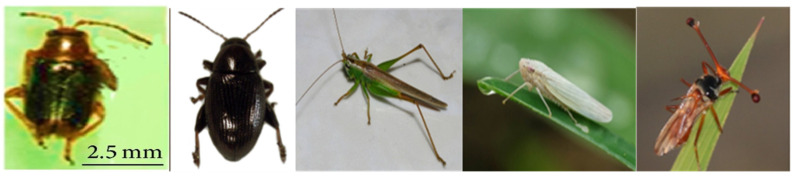
Images of some insect vectors of RYMV from left to right; (i) *Sesselia pusilla* Credit: [28], (ii) an adult chrysomelid beetle *Chaetocnema paspalae* (observed by Stephen Thorpe), (iii) *Conocephalus longipennis*, (iv) *Cofana spectra* from iNaturalist.canada, (v) *Diopsis longicornis* (example of a leafhopper observed by Manuel Ruedi) [34] Accessed on 25 October 2023.

Among these insect vectors, *Hemipterans* are the most efficient and they have biting and sucking mouthparts. *Coleoptera* and *Orthoptera* on the other hand have chewing mouthparts. Most *Coleopterans* and *Orthopterans* feed non-persistently to transmit RYMV [31,32] whereas others like the *Trichispa sericea* (rice hispid) transmit semi-persistently holding the virus between one to three days [18,32]. Some insect vectors have been presented in Figure 1.

Generally, plant viruses are transmitted in non-persistent, semi-persistent and persistent manners. For non-persistent transmission, after acquiring the virus with their stylet (made up of two canals), which they pierce directly upon chewing infected rice, they transmit the virus almost immediately through subsequent feeding to a healthy plant by using the first canal to draw up and sieve the plant sap whereas the second canal injects the virus particle into the plant [33].

Generally, non-circulative viruses are transmitted in a non-persistent or semi-persistent manner. Non-circulative transmission of viruses is the primary and easiest strategy employed by plant virus vectors. According to [35], with non-persistent transmission, the insect vector slurped the virus throughout feeding and right away it attaches itself to the coating of the cuticle in the stylets. The virus merely hangs on the coating to be rapidly injected into the new host, for a small number of minutes after acquiring. Importantly, non-persistent viruses are picked up within seconds to minutes of feeding and transmitted quickly as well. Consequently, insect vectors that pick up non-persistent viruses upon short feeding times on infected plants and can promptly transmit to uninfected plants are known to be highly efficient vectors.

In the case of semi-persistent viruses, the insect vectors need an extended time (minutes to hours) before they can acquire the virus and spread (without a latent period) to healthy plants [36] and the virus is lost when the vector moults. A vector feeding on an infected plant for a very long interval could have reduced effectual acquisition and transmission and sometimes can even end the process of transmission [37]. In persistent transmission of viruses, longer periods of acquisition and inoculation enhance efficiency. Additionally, a latent period is required, and the virus is retained after the moulting of the vector. 

Rice yellow mottle virus has been reported to be spread by mite vectors. These vectors belong to the families *Eriophyidae* and *Tarsonemidae* RYMV [16].

Farm implements, human activities (such as fertilizer application), and plant-to-plant contact can all result in the mechanical transmission of the virus. Additionally, wind-mediated leaf-to-leaf contact, guttation fluids, and irrigation water can spread the virus [26]. Grazing livestock in infected fields spreads the virus by trampling on infected plants and transferring it to healthy ones [38]. Transplanting rice into a field where infected seeds from a previous crop have germinated can also result in the transmission of RYMV to a healthy crop. The virus can further be transmitted between xylem cells through pit membranes [39]. However, no transmission occurred when rice was grown in soil collected from around diseased plants in the field [14].

Additionally, RYMV has the tendency to infect several wild weed species. In the lowlands, insect vectors live off infected wild rice, weed hosts, or self-grown plants, and secondary transmission occurs by wind-mediated leaf-to-leaf interaction, mechanical transmission, or insect vectors [27]. Even though the virus can be found during the development stages of the seed, the virus is not seed-transmitted [40].

Evidence of infections through virus-containing leaf debris, empty rice spikelets, and surface contaminants on rice seeds has been documented [18]. The virus can therefore be transported to new areas far from its origin in rice sacks containing contaminated and partially winnowed rice seed [18]. In Mali, 100% infection rates have been reported in transplanted rice fields, but not in directly seeded fields, indicating a possible connection with infection from the nursery [41]

The virus can remain viable in crude extract for at least 34 days at 27 °C. At 4 °C, it stays infectious in dry leaves for more than 56 days and for several months in frozen leaves. The virus is highly stable in crude sap and can be easily preserved in dry leaves [42].

## 3. Symptomatology and Environmental Influence

**Figure 2:**
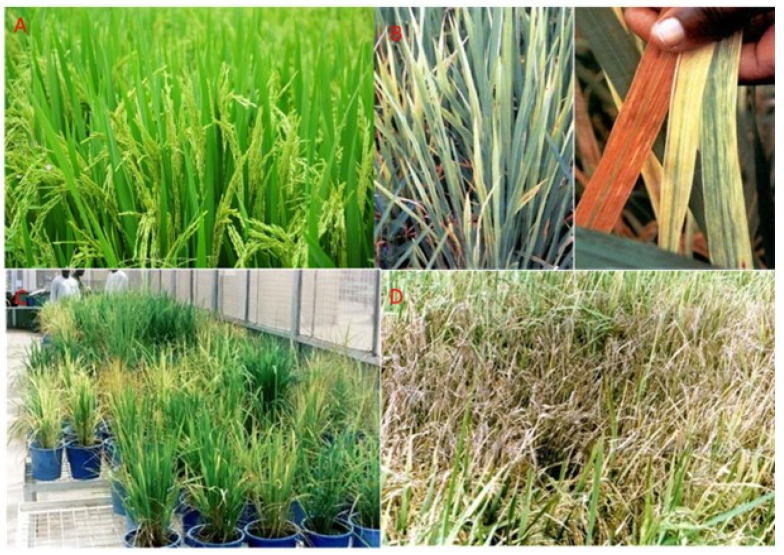
(**A**) An asymptomatic rice plant in the field [45] (accessed on 31 August 2023). (**B**) Leaf coloration symptoms of RYMV; yellow mottle or orange depending on the genotype [14]. (**C**) Yellowing and stunting of RYMV-affected plants. Non-infected plants are uniformly green [14]. (**D**) Field symptoms: a patch of dry leaves of a rice variety severely attacked by RYMV [14].

Symptoms of RYMV mostly are investigated by visual assessment of the coloration of the rice plants. It is easy to distinguish in rice genotypes that are more highly susceptible to RYMV than resistant genotypes [43]. Infected leaves transform from the normal green coloration to yellow stripes with streaks and splotch as the first symptom. Rice genotypes that are more susceptible to RYMV infection have more pronounced symptoms compared to resistant genotypes (Figure 2A–D). Likewise, young plants that are infected display more noticeable symptoms than old, infected plants, and virulent strains exhibit more advanced symptoms than mild strains [44]. Within 14–21 days after initial symptom expression, the spots mature to par with the leaf veins and the color further changes from yellow to orange amidst mottling and wilting for most rice varieties. 

The International Rice Research Institute (IRRI) has technically advanced a standard evaluation system (SES) on a scale of odd numerals from 1 to 9 for the visual diagnostic of symptoms expressed by RYMV. The scale connotes the symptoms descriptions on the basis of the coloration of the leaves as well as the degree of severity of infection as follows: 1—no symptoms; 3—leaves green but with spare dots or streaks and a height reduction of less than 5%; 5—leaves green or pale green with mottling and a height reduction of 6 to 25%, flowering slightly delayed; 7—leaves pale yellow or yellow and a height reduction of 26–75%, flowering delayed; 9—leaves turn yellow or orange, a height reduction of more than 75%, no flowering or some plants dead, modified by [43].

Sometimes, RYMV-affected rice fields might be confused with iron or nitrogen nutrient insufficiency, due to the color change but because RYMV can be spread by vectors, the infected area appears to be in patches whereas nutrient deficiency spans over a stretch or vast area on the field [25]. Symptoms of the disease also include stunted growth, twisting of emerging young leaves, reduction in the total count of the spikelet, decreased tillering, no flowering synchrony, frail exertion of the rice panicle resulting in complete or incomplete sterility [16,46]. There is a drastic reduction in the yield of the plant and the few grains that would develop turn brown. Infected plants might eventually die [26].

Cultivated rice and a few wild grass species are the major hosts of RYMV. The virus includes East African strains [47,48]. The expression of symptoms by RYMV can be strongly influenced by environmental factors such as rainfall intensity, temperature, and relative humidity. These factors affect the virus’s distribution and diversity within a specific area by influencing vector reproduction, host plant growth and susceptibility, transmission and dispersal, survival, and establishment, as well as host–virus interactions [49].

In irrigated or lowland rice farming areas, during the rainy season, RYMV disease prevalence and severity were highest, recorded between 60 and 82%, with a total rainfall of 167 mm and temperatures ranging from 16.8 to 27.7 °C. For instance, in the coastal zone of Tanzania, with a relative humidity of 70.4%, wind speed of 4 km/h, and temperature range of 20 to 31 °C, [50] reported very high RYMV disease prevalence of 82%, and severity of 55%. There is evidence that these weather parameters influence the distribution of RYMV phylotypes within fields and geographical areas. For example, the S4-lm phylotype (Lake Malawian strain) and the S6 strain of RYMV are associated with low temperatures (13.3 °C) and rainfall (13.7 mm), respectively, in Tanzania. The highest RYMV disease prevalence and severity were detected in locations with strong wind conditions, with wind speeds of 9.3 and 18.5 km/h [50].

## 4. Description and Organization of RYMV 

**Figure 3:**
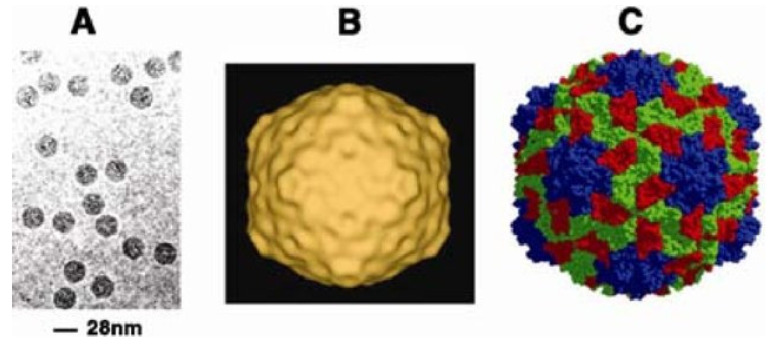
Image of rice yellow mottle virus (RYMV) at different resolutions. (**A**) Electron micrograph of frozen-hybridized native RYMV (Cryoelectron microscopy). (**B**) Three-dimensional surface-shaded density maps of RYMV derived by cryo-EM (55). (**C**) Space-filling model of RYMV generated from X-ray crystallography data. Imaged adopted from [13].

RYMV belongs to the family *Solemoviridae* and the genus *Sobemovirus* [51]. RYMV particles are non-enveloped, rod-shaped virions with a length of approximately 300 nm and a width of about 18 nm (Figure 3). The virion is composed of a single-stranded, positive-sense RNA genome encapsidated by a protein coat. The RNA genome of RYMV is about 7.5 kb in length and encodes several proteins, including those involved in replication, movement, and encapsidation [51]. The virus capsid has 29 kDa coat protein (CP) subunits assembled [15] in a T-3 icosahedral structure. RYMV genome is 4.0–4.5 kb in size with the Malian and Nigerian strains having 4500 nt and 4451 nt, respectively [52]. The virus particle is made up of about 20% RNA and 80% protein with no lipids or carbohydrates [13,53,54]. The icosahedral structure has a molecular mass of 1.4 × 10^6^ Da and it is secured by divalent cations (Ca^2+^), pH-dependent protein–protein interaction, and salt bridges between protein and RNA [55]. The 5′ terminus of the genome of RYMV is a viral genome-linked protein (VPg) in the place of a cap as the 3′ end is not polyadenylated [52]. The lack of cap and poly(A) tail point to the 5′ and 3′ untranslated regions (UTR-s) recompense their function with the 5′UTR bounding covalently to the viral genome-linked protein (VPg) [56]. About 330 nt are in non-coding regions together with a satellite RNA (satRNA; 220 nt; viroid-like RNA), which requires a helper virus for replication yet does not partake in the infection development [57].

**Figure 4:**
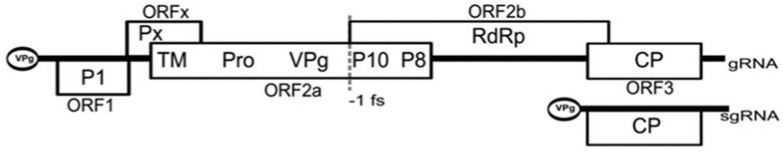
A typical RYMV genome. ORF1, P1: first protein; ORFx, Px: protein “x”; TM: transmembrane domain; ORF2a, Pro-serine protease; VPg, viral genome-linked protein; P10:10 kDa protein; ORF2b, RdRp: RNA-dependent RNA polymerase, CP: coat protein, gRNA: genomic RNA; sgRNA: sub genomic RNA, −1RNA; −1 ribosomal frameshift. Credit: [53].

The coding sequences from 5′ to 3′ consist of five open reading frames (ORFs; Figure 4); ORF1, ORFx, ORF2a, ORF2b, and ORF3 [58]. ORF1 and ORF3 may vary depending on the strain whereas ORF2a and ORF2b are known as the conserved regions [47]. ORF1 codes for PI (first protein), which participates in the suppression of silencing and virus movement. The P1 is required for systemic infection of the plant because it is used for the spread of the virus [59] and helps in suppressing virus-induced gene silencing (VIGS) [60]. ORFs 1, 2a and 2b are translated from the genomic RNA, whereas ORF3, encoding the coat protein, is translated from a sub-genomic RNA. ORFx (Px, protein ‘x’) overlays the 5′ end of ORF2a and continues a little distance upstream of ORF2a. ORFx does not have an AUG initiation codon but expresses initiation of non-AUG; CUG codon by leaky scanning and ribosomal frameshift mechanism between ORF1 and ORF2a initiation codons. The overlapping ORF2a and ORF2b, encrypt the replicational polyprotein which cleaves to give the serine protease together with P10 and P8, viral genome-linked protein (VPg) and the RNA dependent RNA polymerase (RdRP), respectively. VPg is identified to manipulate virulence in opposition to resistance given by the two major genes (*RYMV1* and *RYMV2*) [15,61]. The serine polyprotein protease and the two additional proteins, P10 and P8, have functions that are unknown [54]. 

The RNA-dependent RNA polymerase (RdRp) protein is for replicating the genome as well as for carrying out transcription. ORF3 encodes the viral coat protein (CP) in charge of long-distance cell-to-cell movement, virus packaging, and stability [39,59]. The CP in the RYMV genome is very necessary for full systemic infection to be established in *O. sativa*. Also, virus encapsidation is important before long-distance movement takes effect [13].

An analysis of coat protein gene sequences of a representative sample of 40 RYMV isolates from 11 African countries by [62], revealed a high (~14%) overall level of nucleotide diversity. West/Central African isolates with up to 9% divergence belonged to a monophyletic group, whereas the East African isolates with up to 13% divergence fell into distantly related groups [62]. Sequencing of Tanzanian RYMV isolates resulted in the identification of some as strain S4. Also, three of the isolates, Tz-12-20, Tz-12-22 and Tz-10-36, were clustered as a new group named S4-mk (Mt. Kilimanjaro). The cluster of S4-mk formed a monophyletic group of isolates in strain S4-lv reported from the Tanzanian side of Lake Victoria [63]. According to [64], a comparison with 28 sequences from East Africa showed that they clustered within a new strain named S4et, related to the S4mg and S4ug strains found in the Lake Victoria Basin and Madagascar, respectively. Ugandan isolates of RYMV were reported by [65] to be phylogenetically related to those from the Democratic Republic of Congo, Madagascar, and Malawi but not to RYMV isolates in West Africa. Also, based on coat protein gene and complete genome sequencing, Ghanaian isolates of RYMV almost exclusively belonged to strain S2, one of the strains covering the largest area in West Africa [66].

## 5. Serological and Molecular Typing of RYMV

Diagnosis of RYMV serologically can be performed with the leaf sap from infected rice plants between 14 and 21 days old [18]. The leaf sap obtained can be diluted to the 10th–11th factor with the end point varying on the source of inoculum. As the temperature increases, the number of days for the virus to stay infective reduces. For instance, at 27 to 29 °C, the virus can remain infective for almost 35 days in the raw sap. Furthermore, the virus slowly drops its infecting power at temperatures around 55 °C to 70 °C [44]. On the other hand, as the temperature decreases, the virus can retain its ability to infect for a prolonged period. At a storage temperature of 4 °C, the crude sap can be infective for 84 days, whereas at 9 °C, it can stay for up to 71 days [16]. The virus can equally be preserved in air-dried herbarium pressers, and it can still replicate at a better frequency [18]. 

Immunologically, five main serotypes of RYMV have been typed: Ser1, Ser2, Ser3, Ser4 and Ser5. The West African serotypes are Ser1, Ser2, and Ser3 whereas Ser4 and Ser5 are East African serotypes [13,67]. Molecular typing of RYMV is centered on the sequences of the ORF3 and ORF1 coding for the CP and the movement protein, P1, respectively. Currently, molecular typing of these serotypes identified six strains as S1ca, S1wa, S2, S3, Sa and Sg in West Africa [66] and S4, S5 and S6 in East Africa [68].

These strains have varying pathogenic properties ranging from infection abilities to symptom intensities on rice and wild grasses [69]. RYMV isolates can be grouped into several distinct strains, each with varying levels of virulence. High virulence levels have strains of RYMV which are more aggressive and cause severe symptoms such as significant leaf yellowing, mottling, stunting of plants, and substantial yield losses [70]. These strains are often associated with more severe outbreaks and greater economic impact on rice production. The low-virulence strains are less virulent and may cause milder symptoms or infect plants asymptomatically, resulting in less damage to the crop [62]. The specific identification of the most virulent strains can vary depending on the region and the genetic makeup of the rice varieties grown there. Strains prevalent in West Africa, for example, might show different virulence patterns compared to those in East Africa [62,70].

## 6. Diagnosis of RYMV

Diagnosing RYMV accurately and efficiently is crucial for managing and controlling the spread of the virus in rice fields. Various diagnostic methods are used, ranging from visual inspections to advanced molecular techniques [71]. The choice of diagnostic method depends on available resources, required sensitivity, and specificity, as well as the purpose of the diagnosis (e.g., routine monitoring, research, or investigation of outbreaks).

Initial diagnosis often involves observing the characteristic symptoms of RYMV on rice plants (visual inspection). These symptoms include yellow mottling of leaves, stunted growth, and reduced tillering. However, visual inspection alone is not definitive due to symptom similarity with other rice diseases.

Serological methods such as Enzyme-Linked Immunosorbent (ELISA) are widely used for detecting RYMV due to their sensitivity and specificity [67]. This method involves using antibodies specific to RYMV to detect the presence of the virus in plant sap. There are two main types: direct ELISA uses antibodies directly labeled with an enzyme and indirect ELISA which uses a secondary antibody labeled with an enzyme for detection. ELISA is relatively easy to perform, can process multiple samples simultaneously, and provides quantitative data on virus concentration. However, cross-reaction with other viruses can occur [72], and the method requires proper laboratory facilities and trained personnel.

Nucleic acid-based methods offer more reliable diagnoses with varying levels of sensitivity depending on the method. Dot-Blot Hybridization involves the use of labeled DNA or RNA probes that hybridize with RYMV-specific sequences in plant extracts [61]. The presence of the virus is detected based on the binding of these probes. It is specific and relatively simple to perform but less sensitive compared to PCR-based methods and requires labeled probes and detection systems.

Reverse Transcription Polymerase Chain Reaction (RT-PCR): RT-PCR is a highly sensitive and specific method for detecting RYMV RNA. It involves converting viral RNA into complementary DNA (cDNA) using reverse transcription, followed by amplification to specific DNA sequences using PCR [73]. Conventional RT-PCR amplifies and detects RYMV-specific sequences, providing a definitive diagnosis. 

Real-Time RT-PCR (qRT-PCR): Allows for the quantification of viral RNA in samples, offering more detailed insights into viral load and infection levels. It has high sensitivity, specificity, and the ability to detect low levels of the virus. It also enables strain differentiation based on genetic sequences. However, it requires specialized equipment, reagents, and technical expertise. It is more expensive and time-consuming compared to serological methods [73]. 

Next-Generation Sequencing (NGS) results in High-Throughput Sequencing thus providing a comprehensive analysis of the viral genome, allowing for detailed characterization of RYMV strains and the identification of mixed infections [61]. Offers deep insights into virus diversity, evolution, and population structure but is costly and requires specialized equipment and bioinformatics expertise. 

RYMV exhibits significant variation in virulence among its strains, with some being more aggressive and damaging than others. Therefore, integrated diagnostics combining different diagnostic methods can enhance the accuracy and reliability of RYMV detection. For instance, initial screening with ELISA can be followed by RT-PCR for confirmation and strain identification [26]. This integrated approach ensures comprehensive and accurate diagnosis, which is essential for effective disease management and control.

## 7. Replication and Establishment

Before successful infection can be established, there must be a compatible molecular interaction between the host plant and the virus. The viral infectivity mechanism must be able to overcome the host-plant defenses to ease encapsidation, replication, translation, movement, and assembly [54]. When the genomic RNA of an incoming virion particle enters the cytoplasm of the host cell, the co-translational disassembly mechanism begins uncoating for RNA replication. It has been established that the particles of RYMV can completely disassemble only after initiation of RNA translation [74]. According to [75], the viral replication complex (VRC) replicates the budding complementary negative sense RNA with the initial +ssRNA. The (−) RNAs undergo replication cycles to produce more (+) mRNAs and +ssRNA strands. The (+) mRNAs constructs are translated to produce new viral proteins. The genome is eventually encapsidated to give new virus particles.

New viral particles are conveyed through the plasmodesmata from one cell to the other in the vascular tissues for systemic multiplication [76]. These newly formed viral particles stay in the xylem vessels and are then carried together with intercellular solutes to new cells. The movement protein (ORF1) and coat protein (ORF3) aid in the use of the host protein to modify the plasmodesmata which transports the viral particles by active passage [77]. The size exclusion limit strategy for active transport is deployed for carrying new particles across the plasmodesmata in RYMV [78]. The viral particles of RYMV are usually confined in a variety of host plant cells including the nucleus, vacuole vesicles, mesophyll, bundle sheath, vascular parenchymal cells, epidermis and chloroplast [79,80]. Contingent on the host’s plant stage/age of infection, there could be the transitional (acidic pH dependent) or swollen isoforms (basic pH dependent) more present throughout early infection, as the compact isoforms (Ca^2+^ dependent) are abundant in the course of late infection and these different isoforms influence viral particle stability [80].

## 8. Strategies for Sustainable Management

Integrated pest management (IPM) approaches involving the use of cultural and prophylactic measures have been recommended to control the disease [12]. It has been demonstrated that some traditional agricultural practices, namely seedbed to field transplant and elimination of the wild rice and grasses, which are alternative hosts of both virus and insect hiding places, can influence the agro-ecological modifications on the diversity and the dynamics of the viral populations and consequently RYMV’s prevalence in fields [81]. Some other cultural techniques like the removal of rice ratoons, weeds, and sedges before planting in addition to the destruction of crop residue after harvest are effective in managing the disease. Managing insect vector populations to levels below the threshold in the nursery and in the fields surrounding the nurseries is equally necessary to manage vectors of important viruses [82]. 

Unselective uses of chemicals against the beetle vectors, spraying a light layer of petrol or insecticides on the surface of water in paddy farms to cause the insects to drop into the waters by stretching a cord over the leaves or dropping granulated insecticides in the water of the paddy field making it hostile for the larvae of vector insects are precautionary practical approach [18]. Planting exactly in correspondence to the time and especially in periodic intervals to keep back irrigation water between planting to give a rice-free period and so restrict the buildup of the virus infection and insect vector population [20] are also ways of preventing RYMV. Furthermore, delayed planting up until insect population declines or early transplanting before the outbreak of insect vector, diversifying varieties planted on a single plot or crop rotation, site change for nurseries, sowing from dry seeding by raising nurseries under rainfed instead of irrigation, using a recommended spacing of plants, rouging of infected plants and immediate replanting of healthy plants, reduction in fertilizer application such as urea on the attacked field and flooding of tilled plots while waiting for transplanting in turn to regulate the degree of spread of the disease are all protective measures used to manage RYMV [83]. All these activities aim at disturbing and interrupting the life cycle of the vectors of the disease and improving plant health [84].

However, IPM for RYMV is best achieved when resistant cultivars are used together with cultural and prophylactic methods to give the most efficient control against this virus [13]. This is because resistant cultivars present an extremely economical, ecologically sound, and maintainable endorsed control [85]. Hence, currently, the new trend is breeding for improved resistant cultivars to control RYMV. In developing these resistant varieties or cultivars, a single gene or many genes have been used in a hierarchical approach to obtain partial or highly resistant cultivars [13]. However, the disease is not fully under control, especially in places where the disease occurs in epidemic fraction. Possible sources of resistance to the virus are obtained from screening of *Oryza* germplasm [20] and are engaged as donors to varieties in resistance programs. Resistance in rice to RYMV is addictive in nature and polygenic [86]. No variety is yet commercially available that has both high resistance and other desirable agronomic traits [5]. 

## 9. Basis of Resistance Cultivars to RYMV

Generally, disease-resistant cultivars are developed based on qualitative disease-resistance genes to exhibit monogenic or near complete resistance under the control of major genes [87]. Qualitative disease resistance can be described based on two models: gene-for-gene and the matching allele model [88]. Genetic resistance is the best feasible option for economical and sustainable long-term RYMV management [5]. Gene-for-gene resistance includes initiation of resistance proteins specifically nucleotide-binding domain leucine-rich repeat proteins (NB-LRR), which partake in pathogen attack and beginning of plant defense mechanisms. Nelson et al. [87] related that plants that are resistant usually have genetically dominant (R genes) and most of them encrypt the NB-LRR proteins. Correspondingly, for a virus infection to be completely established, there must be a matching allele for resistance which is given out to susceptibility factors, as in the absence of host factors [89]. At times, the genes expressed in the matching allele stimulate recessive resistance. In the plant host, the recessive host factors comprise eukaryotic-translation initiation factors (eIFs), namely eIF4E, eIF4G and their isoforms. The main form of resistance against plant viruses is the mechanism of recessive resistance by loss-of-function of susceptibility (S genes) [90]. In the study of genetics and molecular biology for management strategies against RYMV, the recognition of resistance genes and quantitative trait loci (QTLs) have been retrieved or genetically sourced from *O. sativa* and *O. glaberrima* [91].

Major resistance genes identified in species of rice in Africa *O. glaberrima* are *Rymv-1*, *Rymv-2* and *RYMV 3* [66]. Molecular mechanisms convening resistance in RYMV from *Oryza* species stem from the monogenic recessive resistance trait *Rymv-1* [92], which was mapped on chromosome 4 [93]. *Rymv-1* was revealed to encode eIF(iso)4G. Before an infection is established, the eIF(iso)4G recruited completely interacts with VPg during pro-viral interaction, whereas in antiviral interactions, mutations caused by contradictions in the eIF(iso)4G impede infection interaction with the VPg of RYMV to give a resistant phenotype. An allelic form, *Rymv1-1* is typical of susceptible varieties, although there are four other allelic variants that are connected to diverse levels of resistance for RYMV. Relatively, *Rymv1-2* was mapped from *O. sativa*, and *Rymv1-3*, *Rymv1-4* and *Rymv1-5*, the three clear-cut resistance alleles were mapped from the indigenous African rice species *O. glaberrima* [94]. These different allelic forms of resistance are known to be a result of conjoining evolution [85]. Chemically, one amino acid difference, a substitution with glutamic acid (E) for lysine (K) at positions E309K and E321K in the middle of the eIF (iso)4G gene, conveys the contrast between *Rymv1-1* and *Rymv1-2* [95], but the resistance in *Rymv1-2* does not give out a very stern immunity; somehow, it permits restricted replication and movement of the wild type of RYMV [96]. 

There have been reports of a breakdown in resistance of *Rymv1-2* because of mutations in the VPg of some RYMV isolates [15]. Nonetheless, substitutions in VPg of RYMV that caused resistance breaks in *Rymv1-2* did not work in *Rymv1-4* plants [94]. Comparably, just a minor group of VPg of RYMV mutants is responsible for resistance breakdown in *Rymv1-3*, and this same subset can overwhelm the resistance in *Rymv1-2* [84]. Even so, virulence isolate is a consequence of mutation in the direct biochemical interaction between the VPg of the RYMV and the eIF(iso)4G of the host rice plant predetermined by *Rymv-1* [15]. Therefore, accessions with *Rymv-1* display complete resistance and they are classified as highly resistant accessions.

Consistent with [97], resistance gene *RYMV3* infers nucleotide binding (NB) and leucine-rich repeat domain protein (NLRs) against the virus programs from the Mla-like clade of NLRs. Hence, the basis for this resistance from *RYMV3* is to oppose the virus by casting a recognition complex with the viral coat protein (CP). 

Quantitative trait loci (QTLs), the region of DNA responsible for controlling a specific trait, have also been identified to express partial resistance to RYMV in some cultivars of rice apart from the resistance induced by *Rymv-1*. Using QTL information in breeding is one of the main applications of marker-assisted selection (MAS). This is the ideal of using markers linked to certain traits (resistance in this case) to select individuals with characteristics of interest. These QTLs have been delineated on the rice chromosomes 1, 2, 7 and 12 [98,99] in dissimilar environments and using distinct resistance criteria, they justify almost 30% of resistance. QTL present on chromosome 12 is concerned with balancing epistasis with a region of chromosome 7 to clarify 36% of virus content [100]. An association flanked by resistance gene with plant architecture and development was proposed by phenotypic connection and colocalization of QTLs. In upland *japonica* rice varieties, this affiliation might clarify, at any rate partly, the average resistance intensity usually detected. On the other hand, on chromosome 12, the QTL of resistance was set to be independent of plant morphology, achieving a chiefly good candidate for introgression into *indica* rice varieties. The consequence of this QTL on chromosome 12 has been used to produce a near-isogenic line, and its interplay with a locus on chromosome 7 has been affirmed in an IR64 genetic background [101]. The durability of genetic resistance is most effectual over time when marshaled in an environmental hotspot for disease development [102]. The stability of a resistant gene is dependent on the pathogen variability, nature of resistance, and environmental factors [103,104,105]. 

Attempts to introgress major RYMV resistance genes and QTLs resistance via conventional breeding methods have been established in vain [106]. The reason is the recessive nature of resistance presented by most RYMV genes. MAS permits the effective selection of RYMV recessive alleles even in the heterozygous state. Selfing or test crossing is not a necessity to detect RYMV alleles in breeding populations; therefore, it hastens breeding development and saves time. Using marker-assisted breeding (MAB) presents the chance to put together more tough and stronger forms of resistance to RYMV by bringing together R and/or S genes with QTLs. Hence, it is vital to understand the influence of each of the RYMV major resistance genes either as solitary or in composite with other resistance genes in advance to distribution. 

As reported by [107,108], using candidate QTLs as combined RYMV major resistance genes or in a single genetic background intend to significantly heighten the strength of resistance because partial resistance retards the breakdown of the major resistance genes. Meaning, for lasting resistance to *Rymv1-2* and other major genes, more QTLs should be detected and incorporated for partial resistance in breeding programs in SSA. Along with it, another way to boost the stability of a major resistance gene is to merge with another major gene particularly those that stop the contact with the viral genome’s conserved domains throughout the infection cycle [109]. As a result, the genetic adjustment of virulent variants to their environments and hosts could be decreased [108]. According to [110], the QTL on chromosome 12 exhibits its partial resistance by detaining the movement of RYMV into the mestome or the bundle sheath cells.

Screening the multigenic families for assessment of genes from eIF4E and eIF4G for possible candidates to code for partial resistance from QTLs against RYMV; three members of the eIF4G were revealed as good candidates. On the other hand, another school of thought on studies in plant–virus interactions discloses that members of the eIF4E family do not implicate resistance [111]. Lately, outside the eIF4E gene, QTL1 was delineated as *Rymv2* [112]. Detailed analysis on *Rymv-2* demonstrates its connection with a regulator of active defense mechanism on the rice homolog of CPR5 (constitutive expresser of pathogenesis-related genes 5), also known as OsCPR5 (*Oryza sativa* CPR5) linked to a point or nonsense mutation in the CPR5-1 sequence [113]. The sequencing of the candidate region showed one nucleotide deletion leading to a truncated and certainly non-functional protein.

The gene codes a transmembrane nucleoprotein to control effector-incited immunity by regulatory cell cycle and defense mechanisms. CPR5 undertakes a conformational switch from oligomer to monomer regarding the activation of immunoreceptors. When this happens, there is a loss of function, which results in the discharge of cycling-dependent kinase inhibitors and the permeabilization of the nuclear pore complex, which sets into motion the basic resistance to many pathogens [114]. Research has proven that focusing on specific host genes for gene modification becomes tricky because of an incomplete number of plant-interacting proteins [97]. Notwithstanding the resistance to pathogens, the mutants with loss of function mostly come out with phenotypes that have undesirable traits for crop improvement [115]. Fortunately, *O. glaberrima* accessions with *Rymv-2* do not exhibit undesirable traits.

The third type of resistance is from a major gene (R) as *RYMV 3*, originating from *O. Glaberrima* and mapped on chromosome 11, which codes for NB-LRR [116]. In plants, representing the most varied and infinite division of R-gene families, the LRR proteins could be unstable amid strongly related plants because of the existence or nonexistence of polymorphism [116]. Considering the molecular background of the NB-LRR gene, virus resistance is articulated in two ways: the hypersensitivity reaction, where the virus is confined at the primary infection site, and the second type, in which there is an extreme reaction where the cell-to-cell movement of the virus is totally halted [117].

An amino acid substitution at positions K779R and A823V on the RYMV3 locus produced two mapped candidate alleles, *NirRYMV 3-R1* and *NirRYMV 3-x*. The third allele, *NIrRYMV 3-y*, is a result of a truncated protein in the LRR area where substitution occurs on the 11th amino acid [115]. *RYMV 3* resistance shows characteristics of maximum reaction where symptoms do not even express after infection [115].

Resistance mediated by the recessive genes (*Rymv-1* and *Rymv-2*) has very few polymorphisms, warranted by conservative selection, resulting in low mutation rates. On the other hand, resistance from the dominant R-gene, *RYMV 3*, is extremely polymorphic with its frequent polymorphism leading to the discovery of quite a few non-synonymous mutations [115]. Selection pressures endorse the evolution of new receptors noticed in almost all LRR genes [116] and this results in offsetting pathogen effectors. Therefore, *RYMV3* has a remarkably high gene variability which may grant resistance to other pathogens [118]. Accordingly, [119] reports that most R genes have been recognized to be race-specific and present resistance to a lone or few strain(s) of a given pathogen. A maximized form of resistance from the recessive *Rymv-1* and the dominant *RYMV3* resistance genes might be able to allow greatly wanted broad-spectrum resistance to RYMV and additional pathogens in rice [119]. The different systems present a detailed understanding of the process by which viruses adjust to plant immunity and provide vital information for the development of ecological resistance contrary to viral diseases of cereals [97].

## 10. Conclusions

The deleterious effects of RYMV have led to the extrapolation of some positive outlook of this disease. Bringing to light the nuisance associated with RYMV resistance management accompanied by the rapid evolution of the virus and resistance-breaking variants observed across SSA, it is important to pay great attention to this disease. The use of resistant varieties involves promoting the cultivation of rice varieties that are resistant to RYMV. Here, promising genes have been mapped out for resistance (*Rymv-I*, *Rymv-2*, *RYMV 3*) for incorporation into farmer/consumer-preferred varieties against the different serotypes and strains of this virus from different biodiversity. Improved diagnosis is also improving knowledge of the disease, which is important for RYMV management. Some regulations on RYMV include disease surveillance and monitoring where there are established robust surveillance systems to detect and monitor RYMV outbreaks. Integrated pest management (IPM) strategy includes combining biological, cultural, mechanical, and chemical control methods. Farmer education and training should be performed regularly to provide instructional programs that will educate farmers on best practices for disease management. Effective disease control measures should be supported by developing and enforcing working policies and regulatory frameworks, including quarantine and restrictions on movement of infected plant materials. This approach would help African countries achieve rice sufficiency more quickly if scientists and breeders continue to utilize these insights for developing improved cultivars, and farmers adopt these improved varieties along with all the IPM strategies.

## 11. Future Prospects

Although there has not been any emphasis on the diversity of the genome of the virus and cultural practices, analysis by in-depth sequencing is underway to describe and contrast exactly the intra-host genetic diversity and structure conditions on the mode of cultivation and the host (wild or cultivated). Even as studies of this nature give a fair insight into the genes and proteins expressed in RYMV–rice interactions, there have not been high throughput genome-based technologies like RNA-seq to investigate RYMV–rice interactions. Elucidating the diverse transcriptomic responses between compatible and incompatible RYMV–rice interactions and explaining the genes included in this procedure, RNA-seq based approaches must be practical. It is necessary to build on the achievement by genomic localization of SNPs related to the major resistance genes by advanced discovery for use in genomic-assisted breeding. Homozygous lines can be technologically advanced through double haploid breeding when combined with MAS for an inexpensive utilization of strong RYMV-resistant varieties. A similar task is demanded to find other important genes and to a greater extent screen host–virus protein interactors to recognize and authenticate supplementary host factors that help or subdue the virus. So, as more susceptible genes are discovered and substantiated to present additional breeding options for RYMV resistance, these genes could be altered with clustered regularly interspaced short palindromic repeats technology (CRISPR-Cas9) to interrupt the interface with viral proteins which could cause these susceptible host plants not to be preferred by the virus, therefore giving resistance.

When any of these interaction factors are picked out, a reverse genetics approach might be expended to ascertain novel host S genes that can be adapted by genome editing to weaken susceptibility. To respond positively to a future RYMV-free/tolerance era, these schemes might issue instant accessions and help create a huge germplasm base and the understanding needed.
